# Alteration in Fluidity of Cell Plasma Membrane in Huntington Disease Revealed by Spectral Phasor Analysis

**DOI:** 10.1038/s41598-018-19160-0

**Published:** 2018-01-15

**Authors:** Sara Sameni, Leonel Malacrida, Zhiqun Tan, Michelle A. Digman

**Affiliations:** 1 Laboratory for Fluorescence Dynamics, University of California Irvine, Irvine, CA USA; 2 Department of Biomedical Engineering, University of California Irvine, Irvine, CA USA; 3 Departamento de Fisiopatología, Hospital de Clinicas, Facultad de Medicina, Universidad de la República, Montevideo, Uruguay; 4 Institute for Memory Impairments and Neurological Disorders, University of California Irvine, Irvine, USA

## Abstract

Huntington disease (HD) is a late-onset genetic neurodegenerative disorder caused by expansion of cytosine-adenine-guanine (CAG) trinucleotide in the exon 1 of the gene encoding the polyglutamine (polyQ). It has been shown that protein degradation and lipid metabolism is altered in HD. In many neurodegenerative disorders, impaired lipid homeostasis is one of the early events in the disease onset. Yet, little is known about how mutant huntingtin may affect phospholipids membrane fluidity. Here, we investigated how membrane fluidity in the living cells (differentiated PC12 and HEK293 cell lines) are affected using a hyperspectral imaging of widely used probes, LAURDAN. Using phasor approach, we characterized the fluorescence of LAURDAN that is sensitive to the polarity of the immediate environment. LAURDAN is affected by the physical order of phospholipids (lipid order) and reports the membrane fluidity. We also validated our results using a different fluorescent membrane probe, Nile Red (NR). The plasma membrane in the cells expressing expanded polyQ shows a shift toward increased membrane fluidity revealed by both LAURDAN and NR spectral phasors. This finding brings a new perspective in the understanding of the early stages of HD that can be used as a target for drug screening.

## Introduction

One of the common characteristic of neurodegenerative diseases is the aggregation of specific proteins that leads to deposits in tissue or subcellular compartments. This is commonly known as protein misfolding or conformational disorder in Alzheimer’s disease (AD), Parkinson’s disease (PD), Huntington disease (HD) and other neurodegenerative disorders^1^. HD is a progressive genetic neurodegenerative disorder with complex pathogenies including protein aggregation and dysregulation of lipid homeostasis. The key event in such pathologies is the conversion of protein to an abnormal conformation that may lead to formation of toxic aggregates. In the case of HD, polyglutamine (PolyQ) aggregation is the hallmark of the disease. In the last decades, there has been an intense research effort to determine the toxicity of the aggregate species as protein aggregation progresses from monomeric to oligomeric and finally a mature inclusion^2,3^. This understanding is relevant since there are currently no effective measures or treatments of HD. Recent research shows that metabolism is also affected as a results of HD and indicates disturbed OXPHOS pathway and a shift of the metabolism toward more free NADH as the disease progresses that implies disruption of glucose uptake in HD cells compared to normal^4^.

Besides glucose, lipid metabolism is also shown to be affected in HD and other neurodegenerative disorders^5–8^. Lipid and cholesterol are important cell plasma membrane components as they control normal cell function. In Alzheimer disease (AD), impaired lipid homeostasis is one of the early event in the disease^9^. The alteration in lipid composition can lead to instability in membrane and synaptic loss in AD^8^. Furthermore, rising evidence indicates that altered lipid metabolism is linked to dysfunction in HD^5^. In particular, research in animal and human indicates that cholesterol metabolism is disturbed in HD^10^. However, the specific alteration remains controversial. In normal condition, sterol regulatory element binding protein, SREBPs, regulate lipid homeostasis by sensing the level of cholesterol in the cell and provides negative feedback in synthesizing more cholesterol. Upon activation, SREBP acts as transcriptional factor and stimulates expression of enzymes that regulate the fatty acid biosynthesis pathway^11^. Some evidence indicates up to 50% reduction in active SREBP in both HD cells and mouse brain tissue^10^. This can be implicated in HD pathogenesis by reduced biosynthesis of cholesterol and fatty acids. Other evidence also show the slower growth of skin fibroblasts of HD patients compared to normal when they are treated with lipid deprived medium^5^. However, other studies suggest accumulation of cholesterol as a results of dysfunction in the transport of cholesterol due to mutant huntingtin interaction with Caveolin-1(vesicles transporting cholesterol through membranes)^12^.

Given that there is a perturbation in biosynthesis of fatty acid or disruption of transport mechanisms of lipid in HD, in this paper we aim to address how membrane fluidity is affected in HD. For this characterization, we used two distinct fluorescent probes: LAURDAN and Nile Red. Both dyes are commonly used for membrane phase characterizations^13,14^. These probes are sensitive to the polarity of the environment since their emission and lifetimes shift toward shorter wavelength and longer lifetime with decreasing solvent polarity^15–17^. On the other hand, in the apolar solvents, the emission of these probes are blue shifted. LAURDAN, 6-dodecanyl-2-dimethylaminonaphthalene, was first developed by Gregorio Weber^18^. It is a common dye used to study membrane fluidity defined as changes in the lipid order as it senses the water penetration into the membrane^19,20^. Nile red (NR), 9-diethylamino-5H-benzo[alpha]phenoxazine-5-one, is another lipophilic stain that has been successfully used to label lipid droplets^21,22^, to characterize total lipid content^23^, and also to study membrane organization^24,25^. NR is an uncharged red phenoxazone dye and its fluorescence emission is altered based on the immediate environmental properties and a change in the dipole moment upon excitation^26,27^.

We used aforementioned dyes and a powerful fit-free approach based on spectral shifts analyzed with the spectral phasor approach to map membrane fluidity in living cells as opposed to the traditional method using normalized ratio-metric assay known as generalized polarization, GP^28–30^. The method and technique presented here has the advantage of analyzing the fluorescently tagged cells, and can detect contribution of multi-components emissions compared to the GP approach. This method was used in two different cell lines, HEK 293 and differentiated PC12, where expanded polyQ HTT exon 1 was tagged with different fluorescent proteins (EGFP and mRuby). We detected membrane fluidity changes by the combining spectral phasor analysis and solvatochromic dyes related to liquid disordered (Ld) and liquid ordered (Lo) phases. Our analysis indicates increased membrane fluidity and shift toward Ld phase when expanded polyQ was expressed. This approach can open up new frontiers to evaluate the efficacy of treatments in HD or other similar neurodegenerative disorders.

## Materials and Methods

Spectral image acquisition was achieved using the 32 channel QUASAR multispectral scanning module on the Zeiss LSM710 laser scanning microscope (Carl Zeiss, Jena, Germany) equipped with Ti: Sapphire laser (Spectra-Physics Mai Tai, Newport Beach, CA) and 63× NA 1.4 oil objective. For each experiment, cells were first visualized using either 488 nm or 561 nm excitation, with respective bandpass filters, to excite either green fluorescent protein (EGFP) or mRuby with the single channel PMT detector. For LAURDAN experiment, we used a 405 nm and 561 nm laser lines to excite LAURDAN and mRuby, respectively. Figure S1 shows a cartoon representation of LAURDAN in the membrane. Nile red was then used separately as a sensor to characterize membrane fluidity in the cells with EGFP labeled HTT protein stained with Nile Red (NR). We employed 2-photon (2-PE) excitation microscopy by setting the excitation wavelength to 950 nm to co-excite NR and EGFP. For all experiments, spectral images were acquired using the lambda mode 32 channels from 416 nm to 728 nm and a bandwidth of 9.78 nm. The frame resolution was set to 256 × 256 pixels and with a pixel dwell time of 12.61μs/pixel. All data were analyzed using SimFCS software available at (www.lfd.uci.edu). All cells were maintained at 37 °C and 5% CO_2_ during imaging.

### Spectral Phasor Plot and Linear Combination Rule

**Figure 1:**
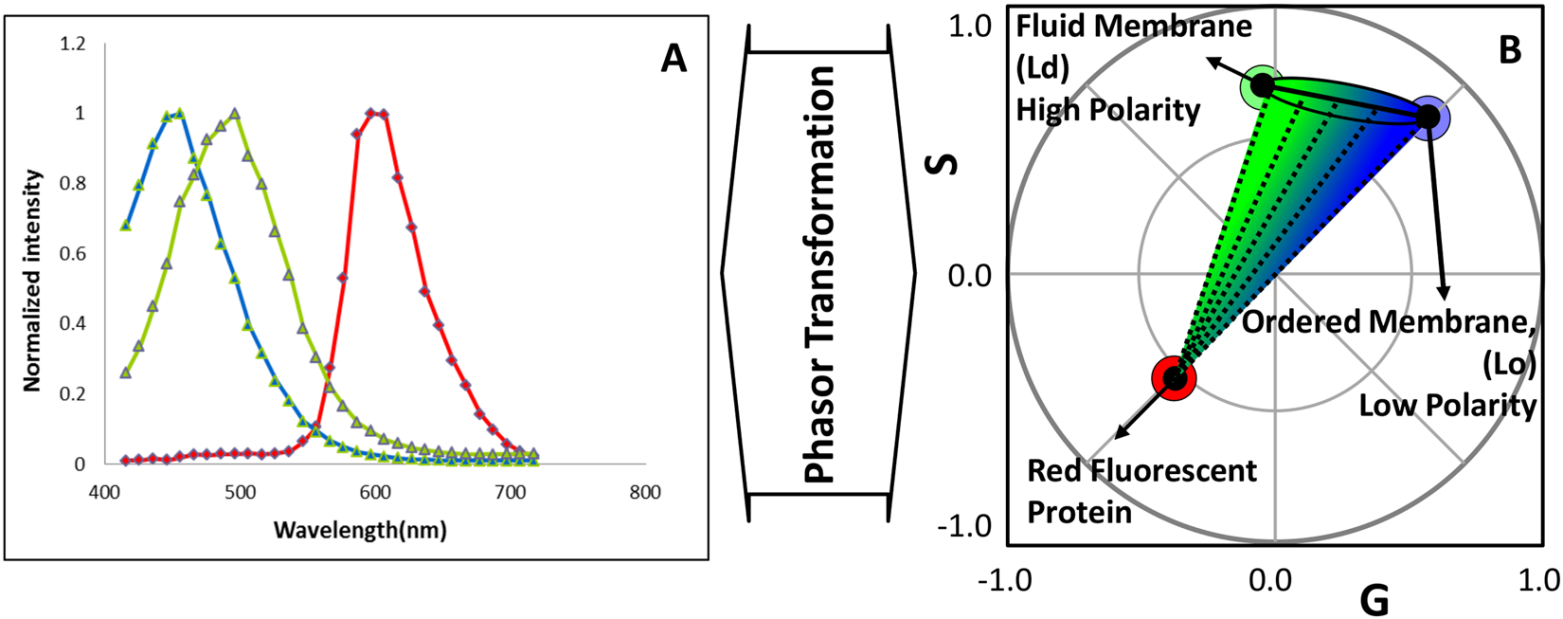
Spectral phasor transformation and the three-component analysis. (**A**) Three spectra for LAURDAN (in a low or high polarity environment, blue and green spectra respectively) and for a red fluorescence protein (RFP). (**B**) The Phasor polar plot with the transformed spectral emissions and a single spot for the emission of LAURDAN in ordered membrane (Lo), fluid membrane (Ld) and a red fluorescent protein (RFP) (blue, green and red dots, respectively). The spectral longer wavelength shift is represented by a counterclockwise shift in the phasor plot from the coordinate (1, 0). The spectra of the LAURDAN are shown in an ellipsoid in 1B which is colored in a gradient from green (high polarity environment) to blue (low polarity environment). If LAURDAN is coexisting with a RFP in the same pixel, then the pixels should fall in a dotted line shown in the picture connecting RFP to LAURDAN trajectory.

Here we employed an elegant way to separate fluorescently tagged protein signal from LAURDAN signal in living cells using a fit-free spectral phasor analysis (Fig. 1).

Spectral phasor analysis is based on the Fourier transformation (shown in equations 1 and 2) of the fluorescence emission spectra (in this case wavelength range is 416 nm to 728 nm) obtained from at each pixel in the hyperspectral image using the 32 channels detector of the Zeiss LSM 710^13,17^. In the equations 1 and 2, I (λ) represents intensity at each spectral step, n is the harmonic number (1 for the data shown in this paper), L is the spectrum range (λ_max_ − λ_min_):

1 $$G=\frac{{\sum }_{\lambda }I(\lambda )\cos (\frac{2\pi n\lambda }{L})}{{\sum }_{\lambda }I(\lambda )}$$

2 $$S=\frac{{\sum }_{\lambda }I(\lambda )\sin (\frac{2\pi n\lambda }{L})}{{\sum }_{\lambda }I(\lambda )}$$

By applying the spectral phasor transformation, we produce a polar plot with a single spot for each pixel. In this case, the emission spectra at each pixel are transformed into two parameters called G and S (real and imaginary component of the Fourier transformation, see equations 1 and 2), and then plotted in a four-quadrant polar plot, called spectral phasor plot, as it shown in Fig. 1B.

Using the phasor approach, we can resolve small spectral shifts on LAURDAN labeled cells that are induced due to changes in the membrane microenvironment even in the presence of fluorescent proteins. The phasor is calculated over a finite wavelength range. The position of the phasor coordinates will change if the emission spectra is truncated as shown in the emission spectral for (Lo and Ld) LAURDAN (Fig. 1A). In the calculations of the Fourier transformation, we used spectra data of a specified wavelength range (416–728 nm). Since all the data has the same wavelength emission range, the comparison between all the experiments are valid. However, if the wavelength emission range is changed the data are no longer comparable. More importantly, the rule of the linear combination of the phasors is not affected by the truncation.

**Figure 2:**
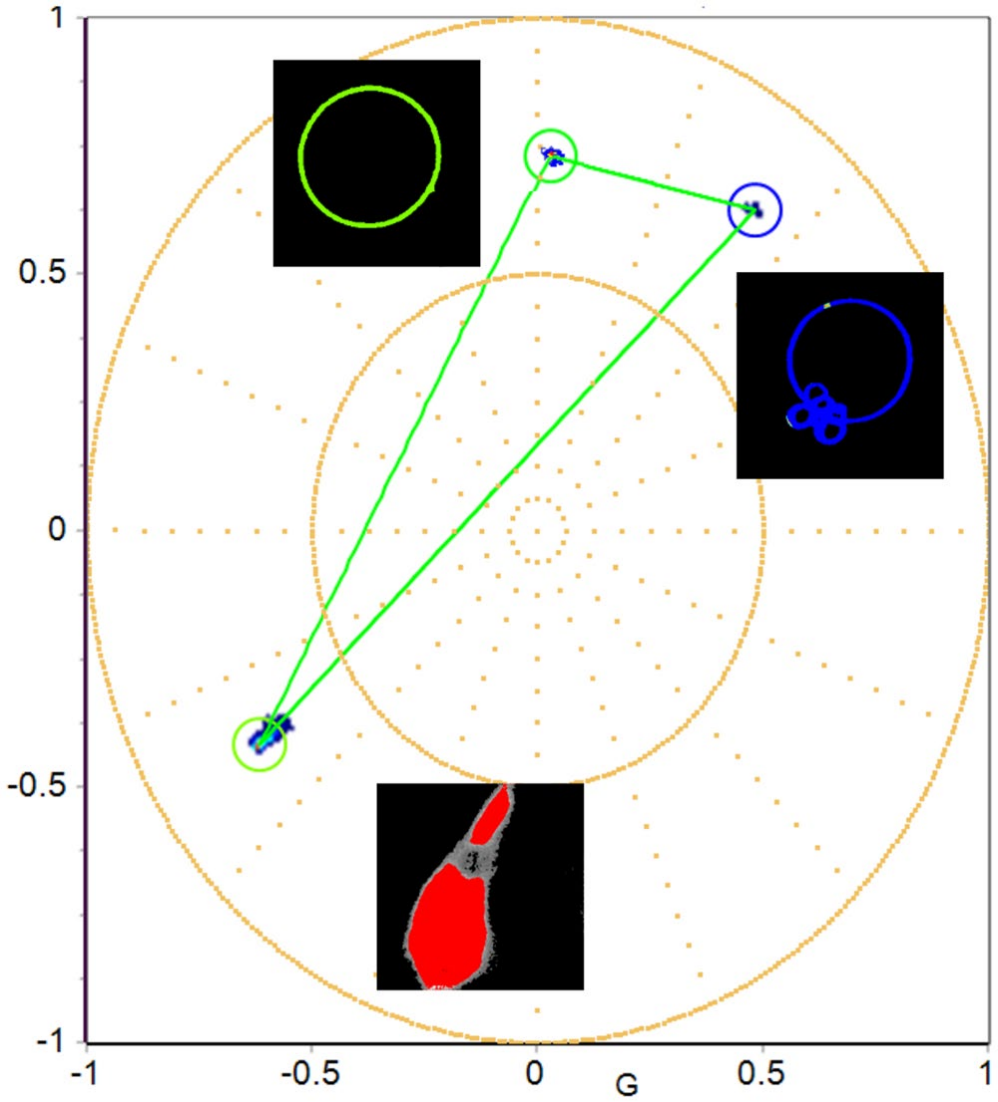
Three component analysis depicting LAURDAN fluorescence emission in Ld/Lo in GUVs and mRuby expressing cells (differentiated 97QmRuby). The framework for the three-component analysis is done by defining the emission of LAURDAN in the liquid disorder (Ld, DOPC, green GUV) or liquid ordered (Lo, DPPC: Cholesterol, 1:1 molar, blue GUV) membranes, and cells expressing the 97Q-mRuby (cell colored by red cursor).

As it is depicted in Fig. 1B the LAURDAN spectral phasors are shown in an ellipsoid with color changing from blue to green that indicates a low or high polarity environment respectively. In cells stained with LAURDAN, if there is a third fluorescent component in the same pixel indicated by the red dot in Fig. 2B, the position of the phasor at that pixel should fall on the dashed lines that joins the LAURDAN trajectory and the fluorescent protein (in this case mRuby) which defines the linear combination. Using linear combination rule, we can resolve complex combinations and characterize membrane fluidity independent of the spectral contamination due to the other fluorescent labels or fluorescent proteins. In this case, blue indicates liquid ordered membrane (Lo) with highly packed phospholipids, and a shift toward green indicates loosely packed liquid disordered (Ld) phase. The penetration of water molecules in the membrane directs the dipolar relaxation of the LAURDAN. Due to the complex deexcitation mechanism of LAURDAN, the spectral phasor analysis provides a better interpretation of fluorescence emission of LAURDAN at the membrane interface compared to classical GP analysis. Note that the LAURDAN spectrum cannot be determined due to the dipolar relaxation effect so that spectral demixing methods cannot be applied in this situation. However, the phasor approach can separate the LAURDAN spectral position from the contribution of the fluorescent proteins by a simple graphical construction.

### Sample Preparation

The stabilized inducible cell model of HD, previously established Htt14A2.6 PC12 cells, as well as newly generated PC12 cells that inducibly express HTT exon 1 containing either 25Q or 97Q repeats fused at C-terminus to EGFP or mRuby were propagated as previously described^31,32^. For differentiation process, cells were supplemented with reduced levels of fetal bovine serum (FBS, 4% or lower) and plated on poly-L-lysine coated glass bottom dish. Neuronal differentiation was induced using 50 ng/ml NGF (Harlan Bioproducts for Science, Inc., Madison, WI) for 7–10 days. Cells were induced with 5 μM ponasterone A (PA) to induce expression of HTT exon fusion proteins 48 hours prior to the imaging.

HEK 293 (ATCC® CRL-1573™) cells were plated on 35 mm glass bottom dish (MatTek, Ashland, MA) that was previously coated with 3ug/ml fibronectin. Cells were transfected overnight using Lipofectamine 2000 (Invitrogen, Carlsbad, CA) and according to manufacturer’s protocol. Transfection was performed with Httex1p 97Q-EGFP, Httex1p25Q-EGFP (kindly provided by Thompson L.M., UC Irvine) or EGFP alone. Cells were then stained with 1 μM NR and incubated at 37 degree for 5 to 10 min followed by imaging.

LAURDAN and NR were used at final concentration of 1.0 μM in the medium for the cell staining.

For cholesterol depletion study, first cells were grown to 70% confluency. Cholesterol was depleted by Methyl-beta-cyclodextrin (MβCD) extracts (Sigma). Cells medium were aspirated, and cells were treated with Opti-MEM™ I (ThermoFisher) Reduced-Serum Medium buffered with HEPES and total final concentration 10 mM of MβCD. Spectral images were then followed immediately by the treatments (every 2–5 min) for 45 min.

### GUV preparation

Giant unillamelar vesicles (GUVs) were prepared follow the electroformation protocol described by Angelova *et al*.^33^. Briefly, dioleylphosphatidycholine (DOPC) or dipalmitoilphosphatidylcholine: cholesterol (1:1 molar, DPPC:Chol) was prepared at a final concentration of 0.3 mM in chloroform. LAURDAN at 0.5% molar was added to the lipid mixture in chloroform. Then, 4 μL of the organic mixture was applied to the each platinum wire and dried under vacuum overnight. The electroformation chamber was filled with 300 μL of sucrose 200 mM at 50 °C. To keep the temperature constant we used a circulation bath at 50 °C. For the GUV growth, we applied a sinusoidal potential of 2 volts and 10 Hz during 1.5 hours. To detach the GUV from the wire, the frequency of the sinusoidal potential was reduced to 1 Hz for 10 min, and then the function generator and circulating bath were turned off. For the measurements, 50 ml of the GUVs dispersion were transferred to 8-wells bottom-glass imaging chamber containing 300μl of a 200 mM glucose solution. The imaging chamber was coated with 1 mg/ml bovine serum albumin (BSA) solution.

## Results

### LAURDAN Fluorescence

Figure 2 shows the range of LAURDAN fluorescence in DOPC representing Ld phase in the membrane (green vesicle) and DPPC: Cholesterol, 1:1 molar (blue vesicle) representing Lo phase in the membrane combined with RFP forming a theoretical triangle with the area of the green triangle indicating all possible linear combinations.

**Figure 3:**
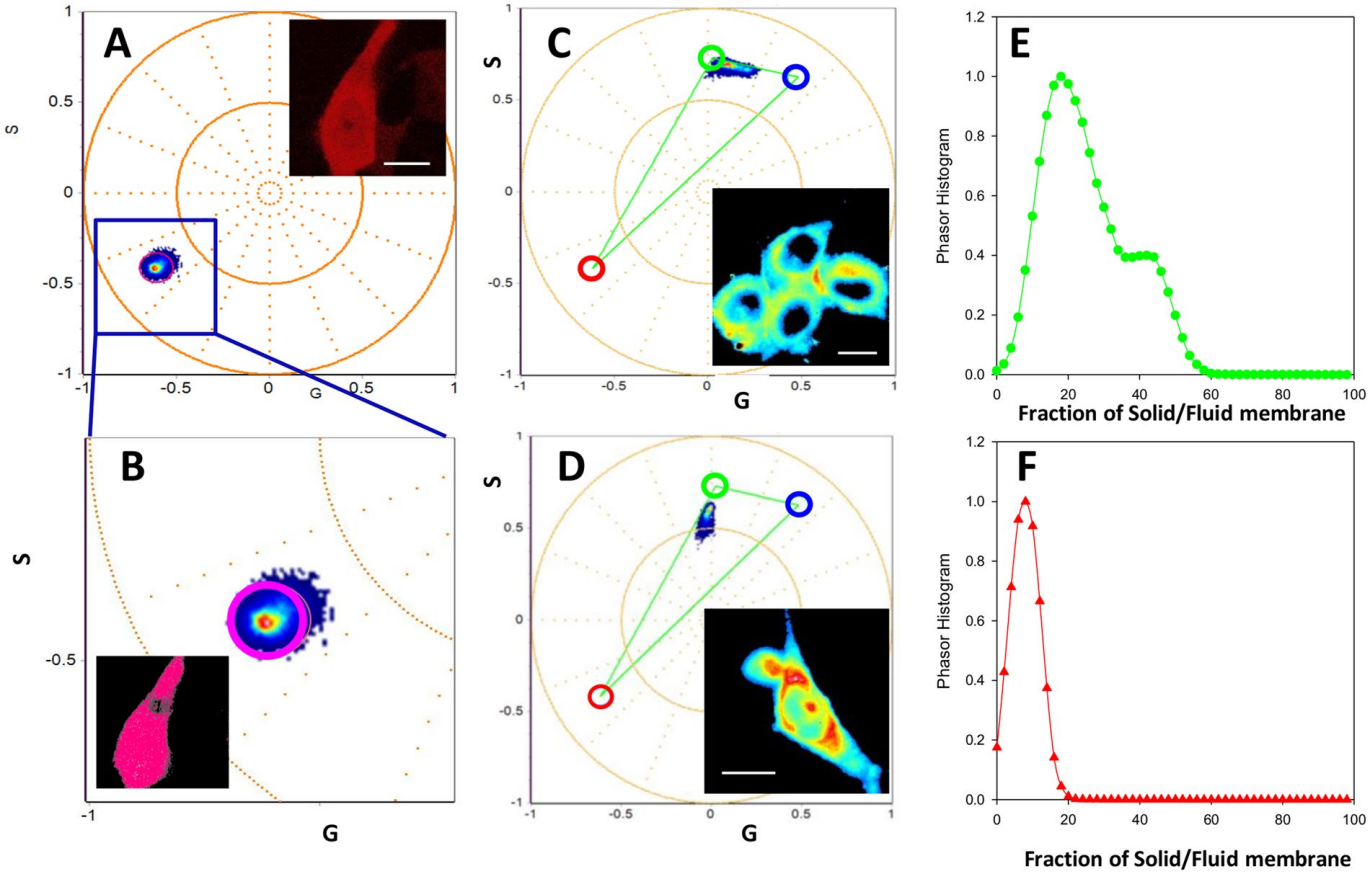
Phasor plot signature for PC12 cell stained with or without LAURDAN. (**A**) Zoomed region from the phasor plot in (**B**) showing a pink cursor highlighting the spectral emission of mRuby of a differentiated PC12 cells expressing 97Q-mRuby in the absence of LAURDAN. Using a pink cursor at the phasor plot we can color code the pixels at the spectral image (see pseudo color image on the top figure). (**C**) Differentiated PC 12 cells stained with LAURDAN in the absence of mRuby with its corresponding fluidity fraction histogram shown in E. The histograms (E&F) indicate the position along the green/trajectory that identifies the fluidity fraction. Here, green cursors means high and blue means low fluidity. (**D**) Differentiated PC12 cells expressing 97Q-mRuby stained with LAURDAN. A trajectory representing all the linear combinations between LAURDAN and the 97Q-mRuby can be seen on the phasor plot. The spectra intensity image is provided on the right side of the spectral graph with the corresponding fluidity histogram shown on (**F**). All Scale bars have a length of 10 μm.

First we identified the spectral phasor position for differentiated PC12 cells expressing 97Q-mRuby. Figure 3A shows a zoomed in region in the phasor plot selecting all the pixels, color coded in pink, corresponding to the phasor position of cell expressing mRuby. Next, we characterized membrane fluidity using the spectral phasor plot in differentiated PC12 cells stained with LAURDAN (Fig. 3C; average intensity of LAURDAN is shown in the inset image). In this case, we used blue and green cursors to identify low to high polar environments sensed by LAURDAN. Then we imaged differentiated PC12 cells expressing 97Q-mRuby (Fig. 3D) to show the phasor position of LAURDAN emission in the presence of 97Q-mRuby. The position along the Lo to Ld phases are shown as a normalized histogram along the blue to green trajectory (Fig. 3E and F). A hypothetical triangle is drawn to depict the combination between the possible lipid phase emissions of LAURDAN with the emission of mRuby.

**Figure 4:**
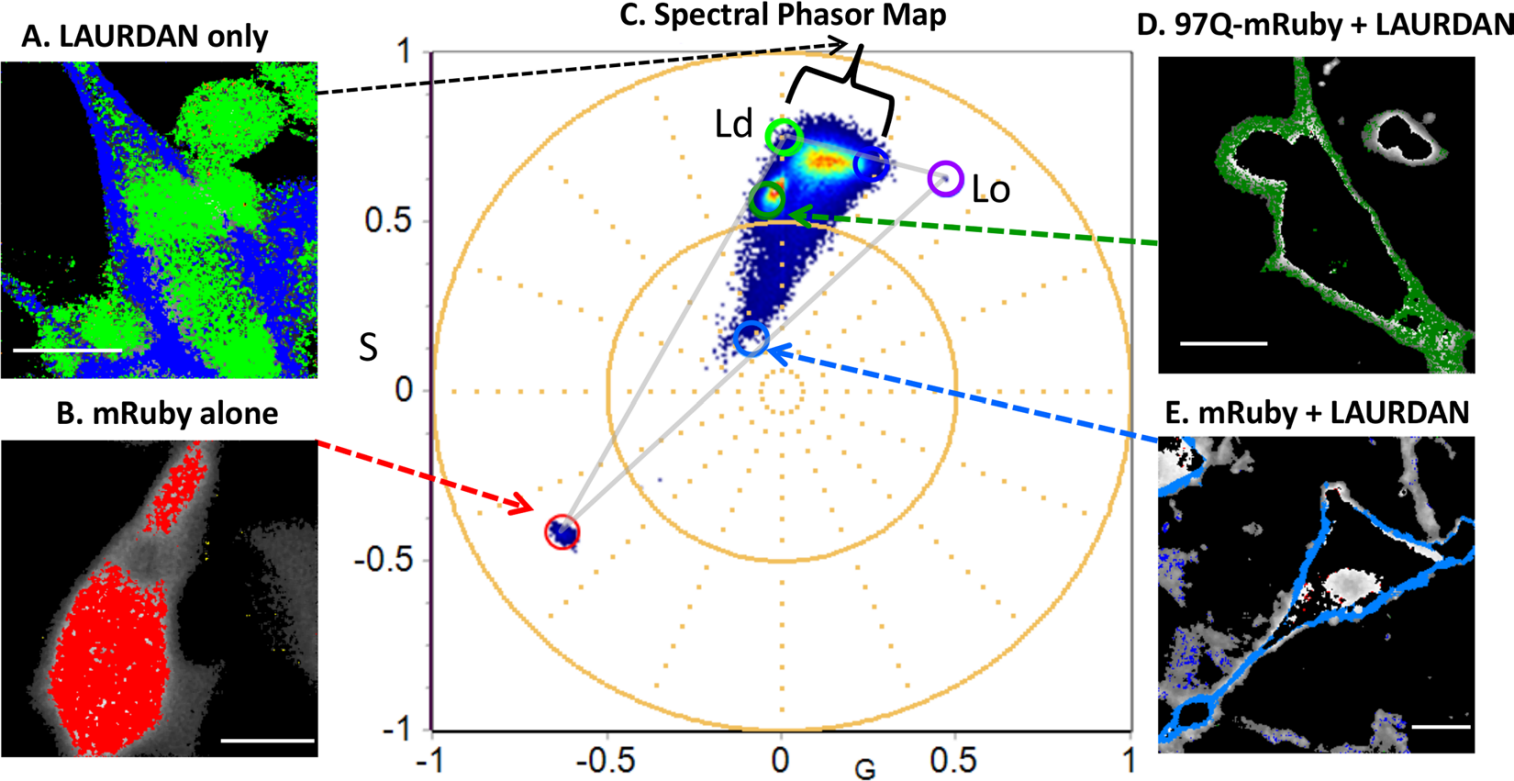
Mapping the emission spectra of LAURDAN and mRuby using spectral Phasor plot in differentiated PC12 cells. (**A**) Differentiated PC12 cells stained with LAURDAN alone are highlighted by the light green and dark blue cursors corresponding to the Ld and Lo phases, respectively. The Ld and Lo spectral emission coordinates were plotted from DOPC and DPPC GUVs (light green and purple cursors; respectively). (**B**) Shows the red pseudo-color image of mRuby alone. (**C**) Corresponding Spectral phasor plot are depicted here in which the light green (Ld) phase and the purple (Lo) phase are connected to the red cursor (mRuby) to make the theoretical triangle of the three pure emission peaks, in gray. As it is shown in D, 97Q-mRuby is characterized with increased fluidity in membrane (shift to Ld phase, highlighted in dark green) compared to the controls (LAURDAN alone and mRuby + LAURDAN, blue color, Lo phase). Scale bar in images are 10 μm.

To characterize the cell plasma membrane fluidity, we either applied a threshold or a cell mask to separate cell plasma membrane and background as it is shown in supplemental material (Figure S2). Figure 4 shows the spectral phasors of differentiated PC12 cells stained with (A) LAURDAN alone, (B) expressing mRuby alone, (D) 97Q-mRuby stained with LAURDAN and (E) expressing mRuby stained with LAURDAN. On the corresponding phasor plot (Fig. 4C), the light green cursor identifies the liquid disorder (Ld) phase and the dark blue cursor highlights the liquid order (Lo) phase, both connecting to the red cursor (mRuby) to make the theoretical triangle, in gray, that encompasses all possible emission species. Figure 4A shows the pseudo-colored highlighted image of the PC12 cells stained with LAURDAN where the dark blue cursor in the spectral phasor plot highlights the pixels identifying the Lo phase and light green cursor highlights the Ld phase. Note that the cell membranes of PC12 cells are highly ordered (dark blue pixels) compared to the inner cellular compartment (light green pixels). As it is shown in Fig. 4D, when we have 97Q-mRuby stained with LAURDAN, we observed a shift towards the liquid disorder phase (Ld) phase (highlighted with dark green pixels) compared to the controls (Fig. 4A and E). When we have both mRuby and LAURDAN presents, the spectral phasor coordinates are aligned in the triangle connecting the fractional contribution of LAURDAN with mRuby.

**Figure 5:**
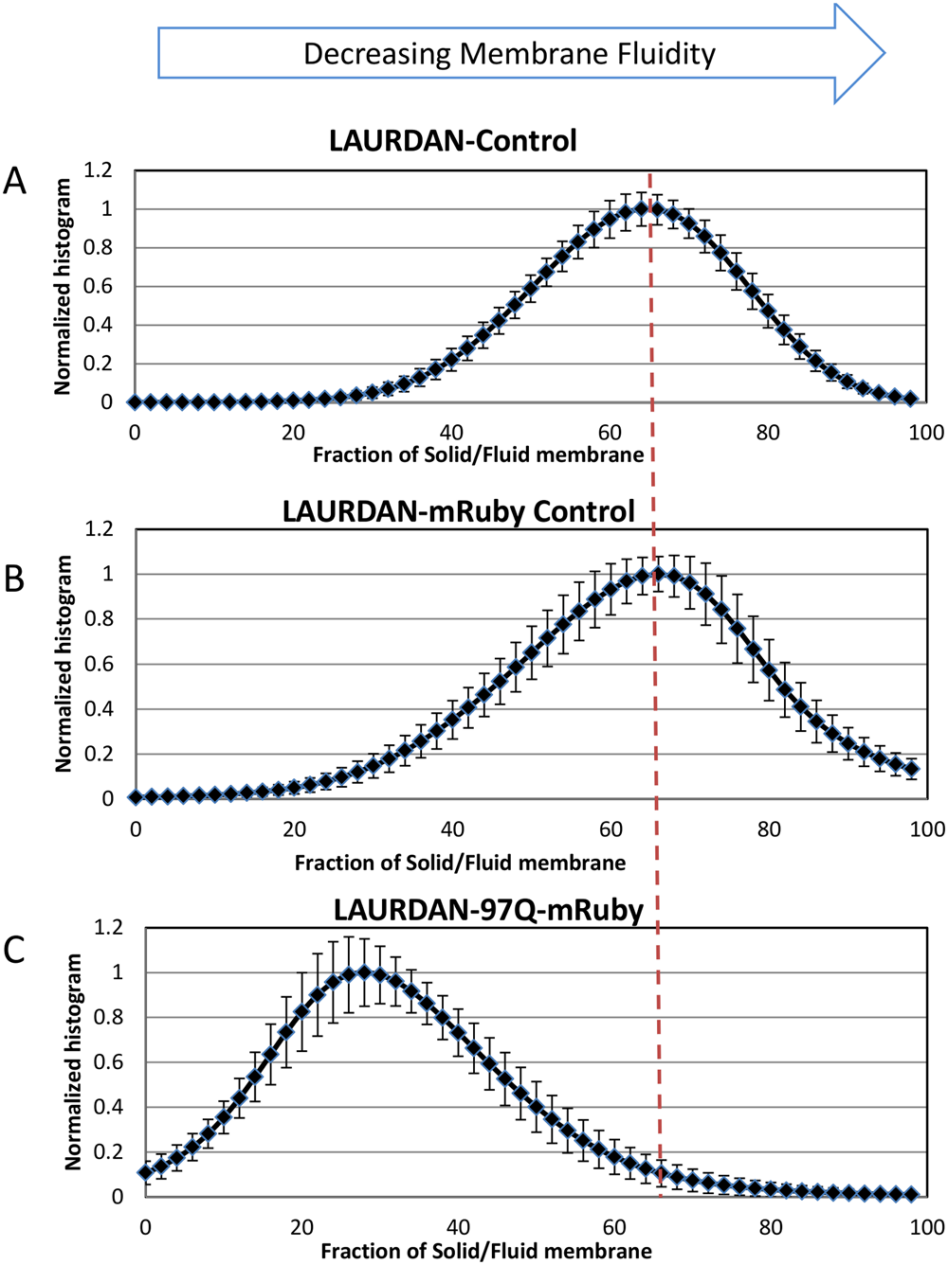
Normalized histograms of the solid to fluid fractions in PC12 cell membranes. (**A**) Cells were stained with LAURDAN only. (**B**) PC12 cells expressing mRuby stained with LAURDAN. (**C**) Histogram of the solid to fluid fraction in cells expressing 97Q-mRuby stained with LAURDAN showing a shift toward increased membrane fluidity (Ld phase) compared to control lines(top two curves, A&B). Data are averaged for N = 25 for LAURDAN-Control, N = 19 for LAURDAN-mRuby-CTR, and N = 16 for 97QmRuby, N = number of the cells, standard deviation are represented with error bars.

To further characterize the membrane stained with LAURDAN, we plotted the normalized histograms of the solid to fluid fractions in PC12 cell membranes along the line between green to blue cursor (Fig. 5). When the phasor position of mRuby is presented together with LAURDAN, the mixture of the possible components will fall inside the theoretical triangle that connects the green/blue cursors and the red cursor. We defined the position along the green (Ld) and blue (Lo) trajectory as a fluidity fraction. Supplemental Figures S3 and S4 shows the histogram of the fraction of solid to fluid phases on the membrane obtained in the differentiated PC12 cells(control line) and in differentiated PC12 cells expressing 97Q-mRuby, respectively.

Figure 5 summarizes the results obtained with LAURDAN. As it is depicted in this summary graph, 97Q-mRuby histogram is shifted toward increased fluidity in the membrane (Ld phase) compared to the cells with LAURDAN alone and LAURDAN-mRuby (controls).

**Figure 6:**
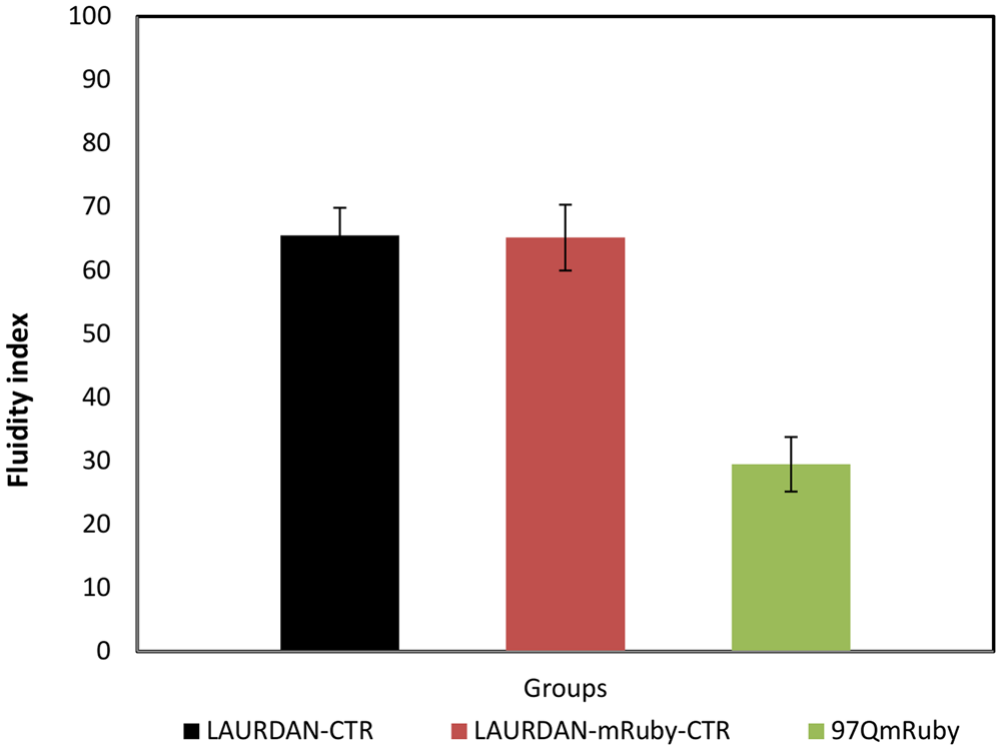
Fluidity index. Cells expressing 97Q-mRuby have a decrease in membrane fluidity index indicating increased membrane fluidity compared to controls.

We have also further characterized the histograms shown in Fig. 5 by analyzing the shift in the center of mass, termed it as fluidity index, where 0 corresponds to the extreme red spectrum (fluid) and 100 corresponds to the extreme blue emission (rigid). The axis is divided linearly according to the linear combination between the Lo and Ld phasor coordinates. Figure 6 shows an increase shift in membrane fluidity index for the membrane of PC12 cells expressing 97QmRuby.

### Nile Red Fluorescence

To confirm if the expression of the 97Q was sufficient to cause a spectral shift in the plasma membrane, we expressed 97Q with EGFP and used another fluorescent probe, Nile Red, which has also shown to be sensitive to the polarity of the lipid environment. NR has an emission peak at 633 nm in Phosphatidylcholine (PC) vesicles and an emission spectra at 621 nm in (1:1) PC:Cholesterol vesicles^22^. For the spectral analysis, we determined the fractional contribution of liquid ordered (Lo) versus liquid disordered phases (Ld). The emission distribution in each pixel in the image is color coded with two different pseudo-colors within the phasor plot: yellow corresponding to low fluidity and blue corresponds to high fluidity in the membrane.

Similar to the experiment with LAURDAN, we applied linear combination properties of the phasor plot to characterize membrane fluidity. Supplemental Figures S5 and S6 display the condition when green fluorescent protein with or without HTT is expressed in HEK293 cell (Figure S5) and differentiated PC12 cells (Figure S6). NR was used to stain the cell and characterize lipid order. In this situation, we identified the location of EGFP tagged protein in the spectral phasor, recognized by the cursor shown in green. Then, we calculated the fraction of the contributing pixels in the lines connecting the protein (green cursor) to the high/low polarity environment for the NR probe (cursors shown in blue and yellow, shown in Figure S6). Supplemental Figure S5, confirm that HEK293 cells expressing 97Q-GFP are shifted toward blue in the yellow/blue trajectory that indicates increased membrane fluidity (Ld phase).

Similar results indicate a shift toward higher polarity sensed by NR in 97Q-GFP indicating fluid membrane in PC12 cells expressing 97Q-GFP compared to 25Q-GFP (control) (Supplemental Figure S6). Supplemental Figure S7 summarizes the results shown in bar graph as a normalized fraction of color contribution obtained in this section. As it is shown in the bar graphs, control cells have a higher fraction of yellow color (Lo phase) indicating highly packed membrane. However in the case of 97Q-GFP, we have higher fraction of blue color (Ld phase) indicating the shift toward higher polarity and increased membrane fluidity.

### Acute Cholesterol Depletion Influences Cell Membrane Fluidity

To correlate the effect of polyQ on cholesterol depletion and modulation of the cell plasma membrane fluidity, cholesterol was depleted by methyl-beta-cyclodextrin (MβCD) extracts (Supplemental Figures S8 and S9). Cells were treated with 10 mM of MβCD that is well characterized to deplete cholesterol in the membrane^34^. Previous study indicates decrease of up to 70% of cellular cholesterol when cells were treated with 10 mM of MβCD for 10 min^35^. Following the treatment with MβCD, we characterized the LAURDAN stained HEK293 and PC12 cells by spectral time-lapse imaging for 45 min. Similar to the shift we observed with 97Q, the shift in membrane fluidity was observed as early as 4 min in HEK293 cells and 2 min in PC12 cells indicated by a shift from a membrane fluidity index of ~70 to ~15. Figures S8 and S9 summarizes the results obtained here in terms of a shift in the fluidity histogram’s center of mass analysis.

## Discussion

While numerous studies indicate reduction of cholesterol and biosynthesis of fatty acid in HD^10,36,37^, other researchers show interaction of mutant huntingtin with Cav-1,a major cholesterol transporter protein, which results in accumulation of cholesterol in neurons^12,38^. Rising evidence also suggest the role of Huntingtin as a trafficking protein^39–41^. This suggests that there is a problem with the lipid homeostasis in HD. In this paper, we aimed to investigate possible modulation of cell membrane fluidity in HD. For this purpose, we used hyperspectral fluorescent microscopy together with the spectral phasor analysis. Two distinct fluorescent dyes (LAURDAN and NR) were used to characterize changes in the fluidity of plasma membrane of the cells affected by the expanded polyglutamine protein. LAURDAN in particular has been well characterized in the literature and shown to be sensitive to the lipid order phases due to the lipid composition and the amount of cholesterol present in the membranes^42,43^. The shift we observed in membrane fluidity measured in the HD cells when expanded polyQ was expressed may reflect depletion of the cholesterol on the membrane^44,45^. We have further verified this by depleting the cholesterol in cells using MβCD. The results show a sharp shift in emission spectra of LAURDAN towards a longer wavelength. This suggests that there may be a possible correlation between the aggregated state of expanded polyQ and its lipid interaction at the membrane. Further studies in this area will need to be done to elucidate the role of the expanded huntingtin protein and cholesterol regulation.

Our results show a clear shift in membrane fluidity of HD cells from a highly packed membrane, liquid ordered, to loosely packed membrane, liquid disordered phase. This shift in fluidity may be implicated in HD pathogenesis, and can also be reconciled with following observations: First, research indicates morphological and mechanical changes in the synthetic lipid membrane due to the interaction of huntingtin aggregates with the membrane^46^. This implies that that physical interaction of huntingtin aggregates can contribute to the membrane fluidity of the cells. Second, the cytotoxic entities of polyQ aggregates that are not eliminated from the cell can influence the cell membrane. It has been shown that dysregulation of fatty acids disrupts autophagy in the cell, the lysosomal degradation pathway essential for cell survival^47^. This is important as autophagic failure is the hallmark of HD and other neurodegenerative diseases^48^. Failure to induce autophagosome in HD allows for the accumulation of the polyQ aggregate forming many toxic small aggregates and large insoluble inclusions that can affect cell membrane. Finally, modulation of fatty acid can directly modify the structure of the lipid membrane and alter micro-domain structure and organization that can be the origin of altered cell signaling^49^. Not surprisingly, alteration of endocannabinoids (eCBS), a powerful regulator of synaptic function signaling has been reported in Huntington disease and other similar neurodegenerative diseases, and it is also believed that this altered eCBs signaling is due to changes in the cell membrane environment with research focused on therapeutic drugs to modulate the eCBs activity^50,51^. Together, our measurements on the fluidity properties of membranes in HD cells shows for the first time that expanded polyQ results in an increased membrane fluidity which c influence other membrane properties such as lipid domain sequestering, ion transport, receptor dynamics, cell to cell contact, and membrane trafficking^52–54^.

The methodologies described in this paper can be used as a platform for better understating of HD and similar neurodegenerative disorders. It also provides a powerful mean to characterize small changes in the membrane microenvironment. This study demonstrates that changes in membrane fluidity can be used as a biomarker for Huntingtin Disease. In addition, the spectral phasors presented in this article, can also rapidly and non-invasively screen for the efficacy of lipid membrane therapies.

## Conclusion

Cell membrane degradation, and defects in membrane trafficking is the hallmark of many neurodegenerative diseases. Here, we have characterized the fluidity in the cell plasma membrane of the HD cells using two distinct membrane probes: LAURDAN and NR in conjunction with spectral phasor analysis. Our investigations indicate alteration in plasma membrane homeostasis that is relevant as it can play a role in cell death, and also can affect control mechanism of ions and molecular transport. We observed a sharp shift using spectral phasor analysis in LAURDAN emission toward longer wavelength indicating increased fluid membrane in HD in both HEK 293 cells and differentiated PC12 cells. As previously discussed, increased membrane fluidity observed here can be related to the observation due to alteration of biosynthesis of fatty acid or disruption of transport mechanism of lipid in HD. In addition, mHTT toxicity through physical or biochemical means can also result in the cell membrane defects reported here. Such alternation in membrane fluidity can be used as a HD biomarker and also for future drug discovery.

## Electronic supplementary material

Supplementary Figures
