# Publisher Correction: Alteration in Fluidity of Cell Plasma Membrane in Huntington Disease Revealed by Spectral Phasor Analysis

**DOI:** 10.1038/s41598-018-24031-9

**Published:** 2018-04-11

**Authors:** Sara Sameni, Leonel Malacrida, Zhiqun Tan, Michelle A. Digman

**Affiliations:** 1 Laboratory for Fluorescence Dynamics, University of California Irvine, Irvine, CA USA; 2 Department of Biomedical Engineering, University of California Irvine, Irvine, CA USA; 3 Departamento de Fisiopatología, Hospital de Clinicas, Facultad de Medicina, Universidad de la República, Montevideo, Uruguay; 4 Institute for Memory Impairments and Neurological Disorders, University of California Irvine, Irvine, USA

Correction to: *Scientific Reports* 10.1038/s41598-018-19160-0, published online 15 January 2018

The Supplementary Figures were omitted from the original version of this Article. This has been corrected in the PDF and HTML versions of the Article.

