# Supplementary material for: Alteration in Fluidity of Cell Plasma Membrane in Huntington Disease Revealed by Spectral Phasor Analysis
Source: Sci Rep. 2018 Jan 15;8:734. doi: 10.1038/s41598-018-19160-0 (PMC5768877; doi:10.1038/s41598-018-19160-0)
Supplement: Supplementary file 1 — Supplementary Figures [file 41598_2018_19160_MOESM1_ESM.pdf]

Appendix

Alteration in Fluidity of Cell Plasma Membrane in Huntington Disease Revealed by Spectral Phasor Analysis

Sara Sameni[1,2], Leonel Malacrida[1,2,3], Zhiqun Tan[4], and Michelle A. Digman[1,2*]

[1]Laboratory for Fluorescence Dynamics, UC Irvine, CA,USA, [2]Department of Biomedical Engineering, UC Irvine, CA,USA, [3]Departamento de Fisiopatología, Hospital de Clinicas, Facultad de Medicina, Universidad de la República. Montevideo, Uruguay, [4] Institute for Memory Impairments and Neurological Disorders, University of California, Irvine.

*Correspondence to mdigman@uci.edu

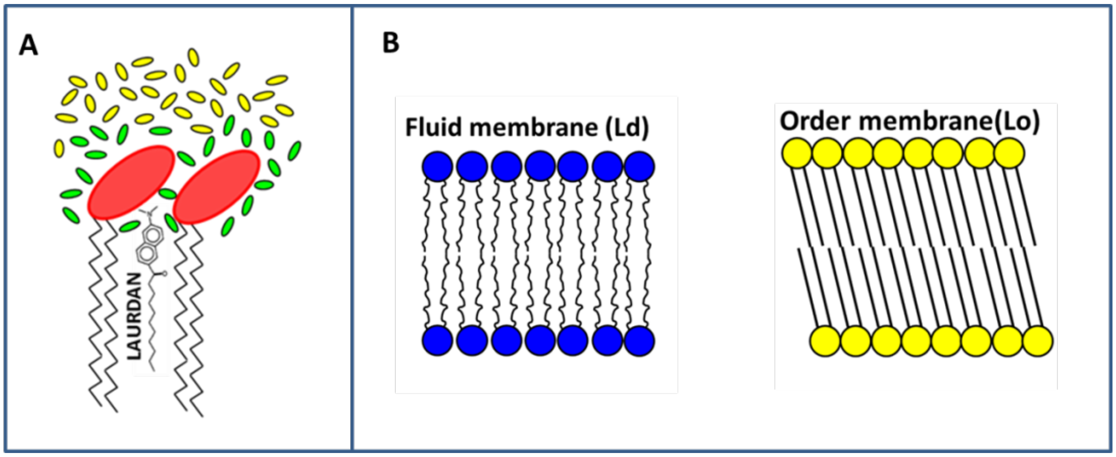

Figure S1: Schematic of LARUDAN in the membrane. A) schematic figure of plasma membrane is depicted with fluorescent membrane probe LAURDAN with the emission sensitive to the polarity of the membrane B) schematic of cell membrane fluidity ; fluid membrane (Ld) is depicted in blue compared to ordered membrane(Lo) depicted in yellow.

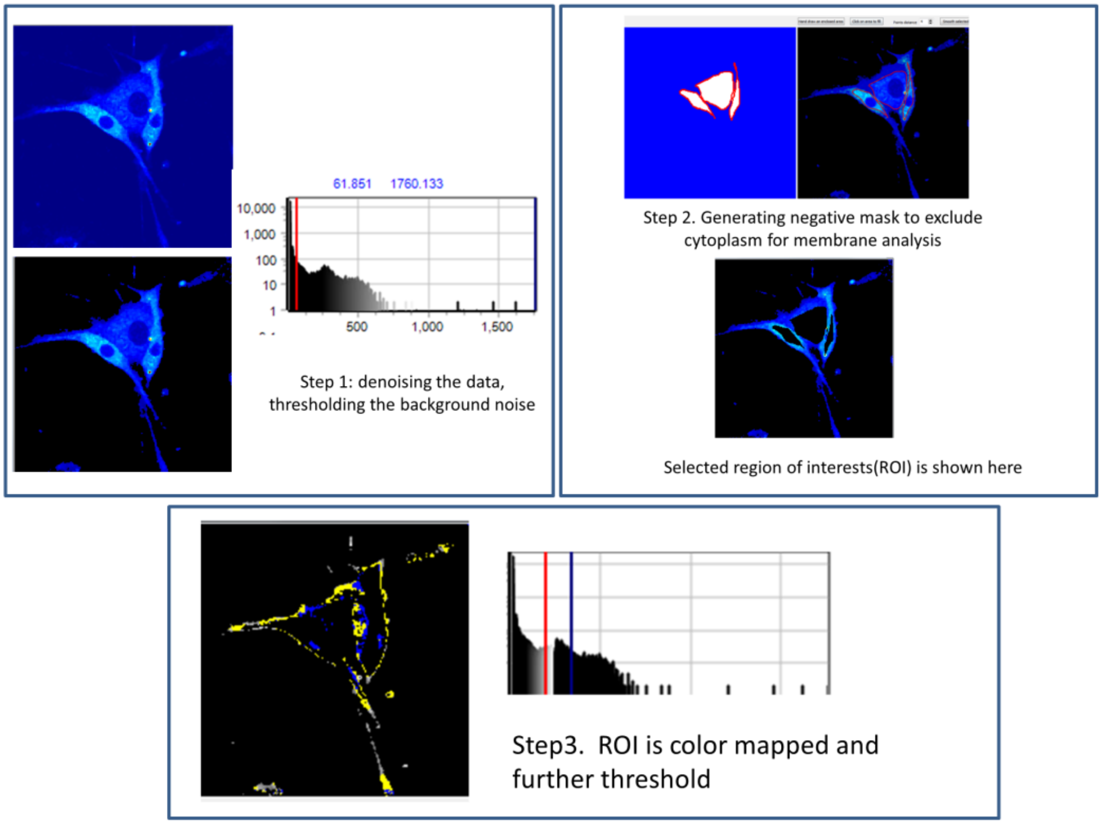

Figure S2: The process of generating membrane masks. First image is threshold to remove the background, and then negative masks are made by excluding the cytosolic region to isolate the membrane. Selected ROI are then color coded on spectral phasor and then further threshold to generate the optimum masks.

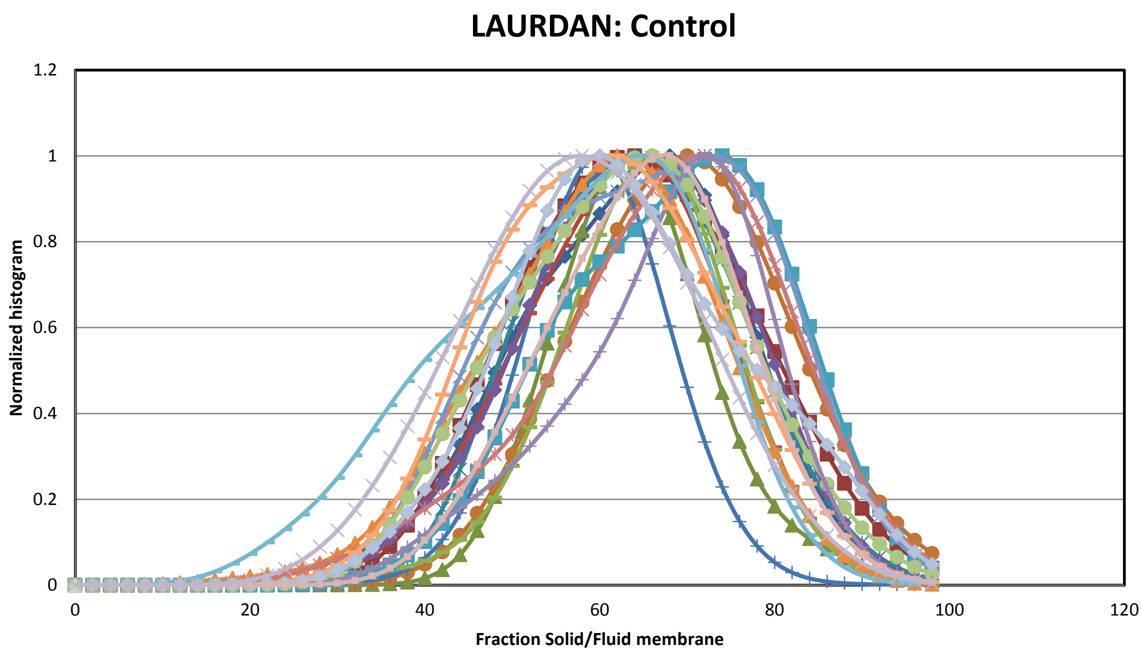

Figure S3: Normalized histogram of the fraction of solid to fluid contribution with LAURDAN in the masked membrane (differentiated PC12 cells without PA). Histogram shows the position along the green/blue trajectory in the membrane as a fluidity fraction which indicates shift toward blue trajectory (Lo phase), each colored curve corresponds to different cells.

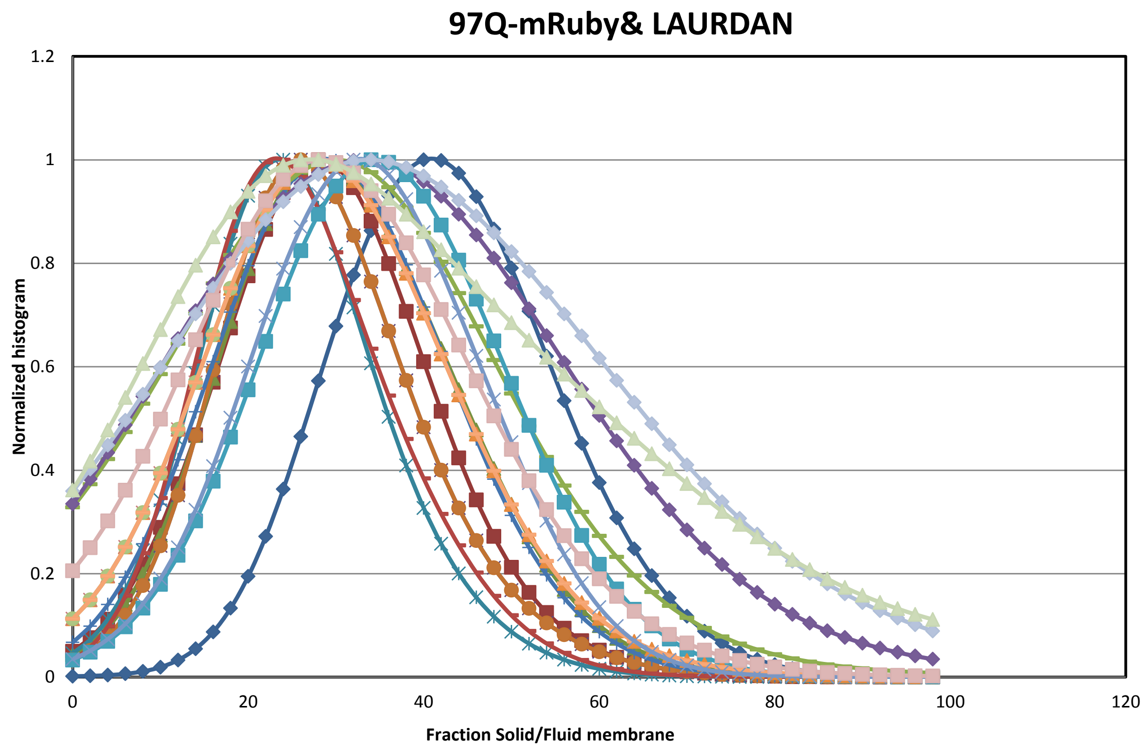

Figure S4: Normalized histogram of the fraction of solid to fluid contribution of cells expressing 97Q-mRuby stained with LAURDAN in the masked membrane (expanded PolyQ). Histogram shows the position along the green/blue trajectory as a fluidity fraction which shows the shift towered green trajectory (Ld phase), each colored curve corresponds to different cells.

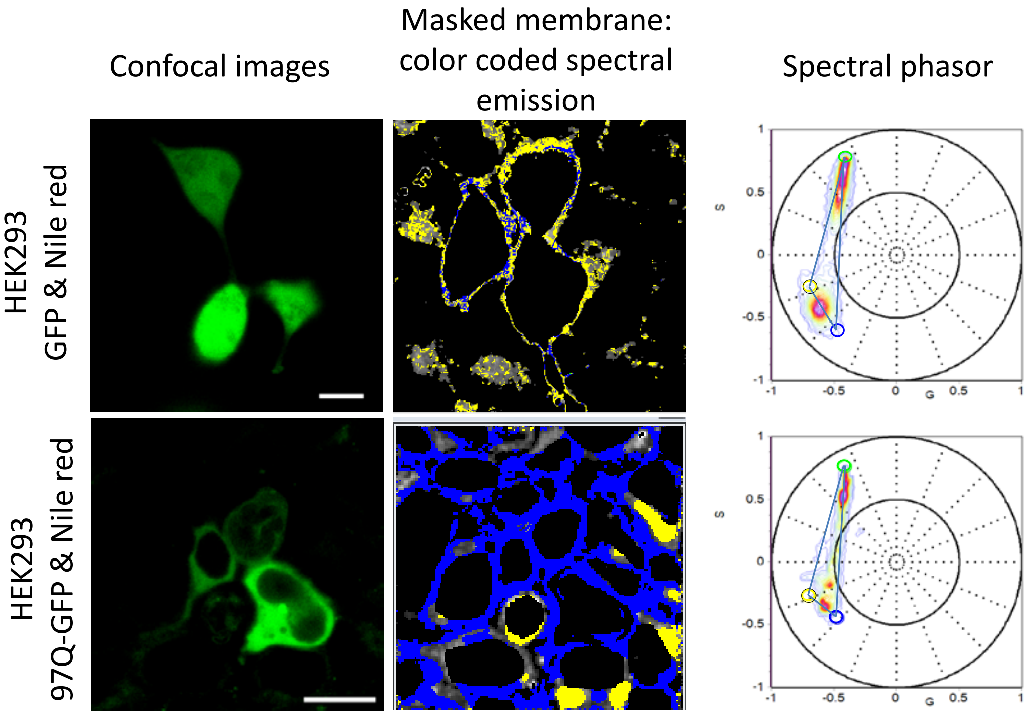

Figure S5: Nile Red and HEK 293 cells. HEK 293 cells expressing 97Q-GFP(bottom panel) and GFP(top panel) that are stained with Nile red are depicted here with the corresponding cell masks generated and color coded with blue and yellow with black outline as it is shown in the phasor plot on the right. The triangle on the graph shows the linear combination rules. As it is presented here 97QGFP is shifted toward longer wavelength indicating increased polarity in NR and increased in the fluidity of the membrane (Ld). Scale bar is 20µm.

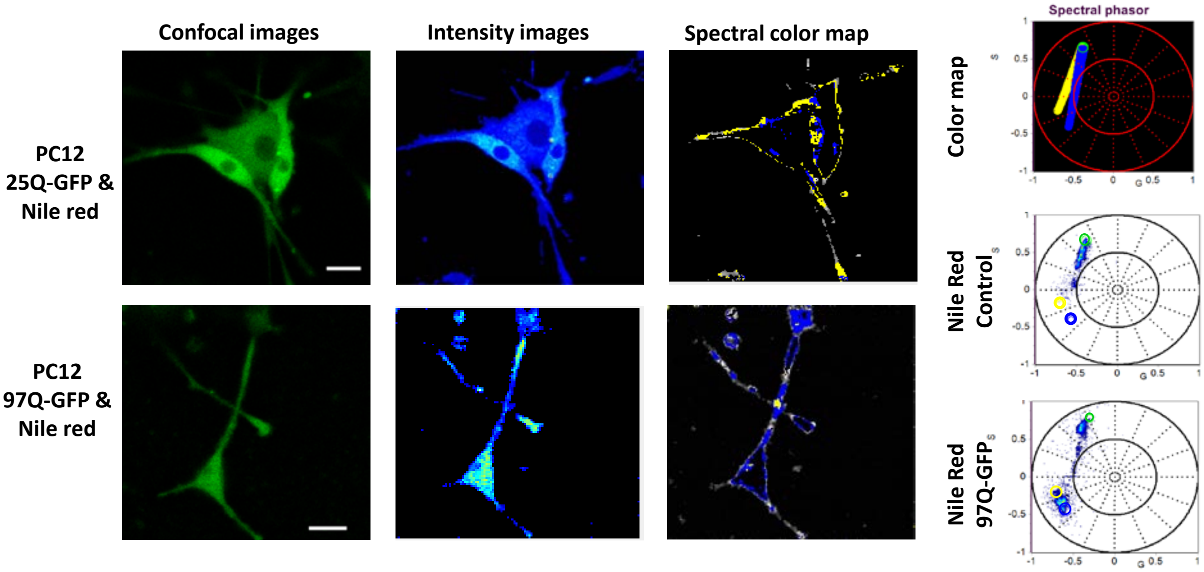

Figure S6: Differentiated PC12 cells expressing 25Q-GFP (control) and 97QGFP are shown here. Color coded membrane masks are depicted next to intensity images. The corresponding spectral phasor plots are shown on the right hand side. The first plot shows how cells are color coded. As it is shown here, there is a shift toward higher polarity for NR in 97Q-GFP indicating fluid membrane. Scale bar is 20μm.

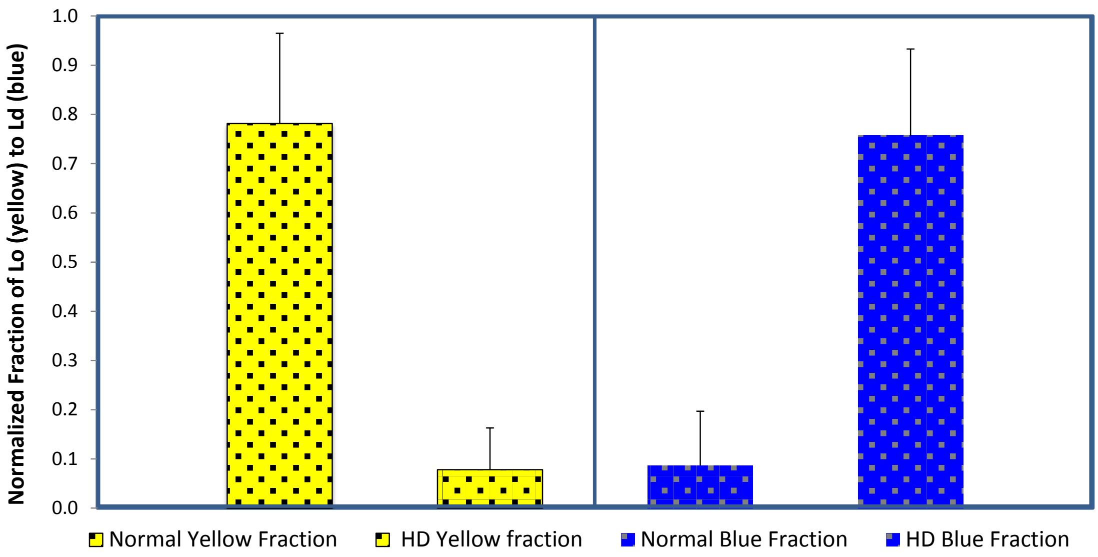

Figure S7: The results obtained using fluorescent probe NR is summarized on the bar graph and by calculating the fraction of color contribution. Yellow bars (pseudo-color) designates fraction of Lo compared to blue bars (pseudo color) fraction of Ld phase in the membrane for N=20 HD (n=10 for HEK293 97Q GFP and n=10 differentiated PC12 97Q GFP, n=number of the cells) stained with NR and N=21 control cells (n=11 HEK293-GFP,n=10 differentiated PC12 25Q GFP, n=number of the cells) stained with NR .Standard deviation are represented with error bars. The graphs indicate shift to high fluidity in the membrane of 97q-GFP cells (Ld phase) compared to control.

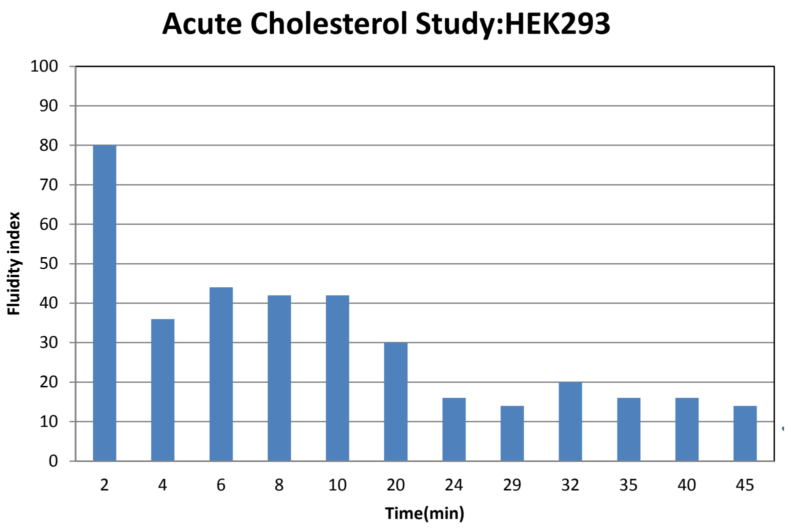

Figure S8: Cholesterol depletion study in HEK 293 Cells. Methyl-beta-cyclodextrin (MβCD) was used to deplete cholesterol in the cell plasma membrane. As it shows here there is a sharp shift in the fluidity index indicating increased fluidity in the membrane in HEK293 cells (n=12).

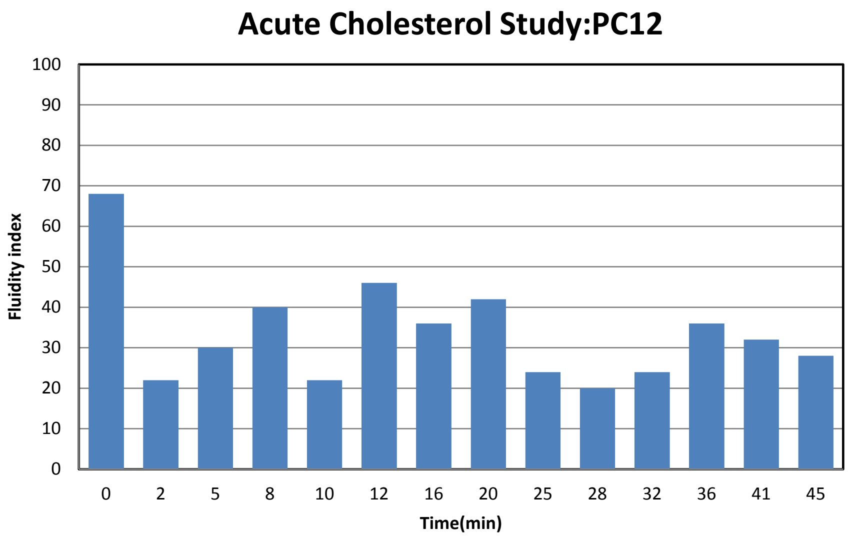

Figure S9: Cholesterol depletion study in PC12 Cells. Similar to figure S8 there is a shift toward increased membrane fluidity as early as 2 min in PC12 cell lines (n=14).
